# A molecular cell biology toolkit for the study of meiosis in the silkworm *Bombyx mori*

**DOI:** 10.1093/g3journal/jkad058

**Published:** 2023-03-13

**Authors:** Youbin Xiang, Dai Tsuchiya, Fengli Guo, Jennifer Gardner, Scott McCroskey, Andrew Price, Eelco C Tromer, James R Walters, Cathleen M Lake, R Scott Hawley

**Affiliations:** Stowers Institute for Medical Research, Kansas City, MO 64110, USA; Stowers Institute for Medical Research, Kansas City, MO 64110, USA; Stowers Institute for Medical Research, Kansas City, MO 64110, USA; Stowers Institute for Medical Research, Kansas City, MO 64110, USA; Stowers Institute for Medical Research, Kansas City, MO 64110, USA; Stowers Institute for Medical Research, Kansas City, MO 64110, USA; Faculty of Science and Engineering, Groningen Biomolecular Sciences and Biotechnology Institute, University of Groningen, Groningen 9747 AG, The Netherlands; Department of Ecology and Evolutionary Biology, University of Kansas, Lawrence, KS 66045, USA; Stowers Institute for Medical Research, Kansas City, MO 64110, USA; Stowers Institute for Medical Research, Kansas City, MO 64110, USA; Department of Molecular and Integrative Physiology, University of Kansas Medical Center, Kansas City, KS 66160, USA

**Keywords:** Lepidoptera, *Bombyx mori*, meiosis, synaptonemal complex, SYCP1, SYCP2, SYCP3, HOP1, PCH2

## Abstract

Meiosis is usually described as 4 essential and sequential processes: (1) homolog pairing; (2) synapsis, mediated by the synaptonemal complex; (3) crossing over; and (4) segregation. In this canonical model, the maturation of crossovers into chiasmata plays a vital role in holding homologs together and ensuring their segregation at the first meiotic division. However, Lepidoptera (moths and butterflies) undergo 3 distinct meiotic processes, only one of which is canonical. Lepidoptera males utilize 2 meiotic processes: canonical meiosis that produces nucleated fertile sperm, and a noncanonical meiosis that produces anucleated nonfertile sperm which are nonetheless essential for reproduction. Lepidoptera females, which carry heteromorphic sex chromosomes, undergo a completely achiasmate (lacking crossovers) meiosis, thereby requiring an alternative mechanism to ensure proper homolog segregation. Here, we report that the development of a molecular cell biology toolkit designed to properly analyze features of meiosis, including the synaptonemal complex structure and function, in the silkworm *Bombyx mori.* In addition to standard homology searches to identify *Bombyx* orthologs of known synaptonemal complex encoding genes, we developed an ortholog discovery app (Shinyapp) to identify *Bombyx* orthologs of proteins involved in several meiotic processes. We used this information to clone genes expressed in the testes and then created antibodies against their protein products. We used the antibodies to confirm the localization of these proteins in normal male spermatocytes, as well as using in vitro assays to confirm orthologous interactions. The development of this toolkit will facilitate further study of the unique meiotic processes that characterize meiosis in Lepidoptera.

## Teaser

Xiang et al. report the construction of a toolkit for the study of meiosis, specifically the synaptonemal complex (SC), in the silkworm *Bombyx mori*. They identify silkworm orthologs for many well-studied SC proteins and develop a tool for finding other meiosis genes in this species. In addition, they report the creation of useful antibodies that recognize the SC. These reagents will provide the tools necessary to study the cell biology of meiosis in this important organism.

## Introduction

Meiosis is essential for gametogenesis in sexually reproducing organisms. In many eukaryotes, meiosis involves the pairing of homologous chromosomes, the building of a proteinaceous structure known as the synaptonemal complex (SC) between homologs, and the exchange of genetic material through the process of homologous recombination or crossing over. These recombination events, marked by chiasmata, are typically considered essential for the accurate segregation of homologs at the first meiotic division. Thus, it is striking that achiasmy (the complete absence of recombination) has evolved repeatedly, with at least 30 independent instances known among animals (Burt *et al.* 1991). In all of these cases, it is the heterogametic sex that lacks recombination (the “Haldane-Huxley Rule”), a pattern that is generally thought to reflect the pleiotropic effects of selection against recombination between differentiated sex chromosomes (Haldane 1922; Huxley 1928; Nei 1969; Hedrick 2007; Blackmon and Brandvain 2017; Stapley *et al.* 2017). In such cases, the evolution of achiasmy can preempt the standard mechanisms of meiotic systems that rely on crossing over to ensure homologous segregation at anaphase I. In the absence of crossing over, alternative and novel molecular mechanisms may evolve to ensure chromosome segregation (Wolf 1994; Zickler and Kleckner 1999).

A classic example of this is seen in *Drosophila*, where the chromosomes of the homogametic (*XX*) females’ crossover, whereas the chromosomes of the heterogametic (*XY*) males do not. A sexually opposite, but parallel, example occurs in Lepidoptera (moths and butterflies), where females are heterogametic (*ZW* or *ZO*) and do not recombine (Sturtevant 1915; Suomalainen 1965; Suomalainen *et al.* 1973; Traut 1977). Female heterogametic achiasmy is also reported in Trichoptera (caddisflies), the sister taxon of Lepidoptera, suggesting it evolved in the common ancestor of these groups (Suomalainen 1966).

In addition to the unique meiosis that occurs in Lepidoptera females, male Lepidoptera undergo 2 types of meiotic processes, only one of which is canonical. Lepidoptera males are homogametic (*ZZ*) and undergo dichotomous spermatogenesis producing regular nucleated sperm (eupyrene) by a canonical meiosis and anucleated nonfertile sperm (apyrene) by an atypical meiosis. Both eupyrene and apyrene sperm are derived from the same primary spermatocytes, and the path they undergo is genetically controlled very early in meiosis. In fact, as early as zygotene stage of prophase characteristics can been seen for whether spermatocytes will produce eupyrene or apyrene sperm. These differences include many of the hallmarks of canonical meiosis. In early meiotic prophase, apyrene spermatogenesis is defective in homologous chromosome pairing, lack the SC structure, and fail to form chiasmata, leading to the final outcome of sperm with an extruded nucleus [reviewed in Friedländer *et al.* (2005)].

Apyrene sperm also differ from eupyrene sperm in terms of the timing of their production in the life cycle in lepidopteran males. Eupyrene spermatogenesis occurs first, beginning in the larval stages. However, apyrene spermatogenesis, which is regulated by Sex-lethal (Sakai *et al.* 2019; Chen *et al.* 2020), does not begin until the second day of prepupation, after which production of eupyrene sperm ceases. By adulthood, 80% of the sperm bundles are apyrene (Cheng *et al*. 2022). While the apyrene sperm may be lacking a nucleus they are nonetheless essential for fertilization (Sahara and Kawamura 2002; Sakai *et al.* 2019). The role of the apyrene sperm in fertilization is not fully understood. In 1 species, it was demonstrated that apryene sperm are required for transport of eupyrene sperm to female storage organs (Sakai *et al.* 2019; Chen *et al.* 2020). However, it is further speculated that apyrene sperm play a key role in sperm competition in many species, and/or provide nutritional value to the female or eupyrene sperm [potential functions for apyrene sperm reviewed in Friedländer *et al.* (2005)].

The silkworm *Bombyx mori* serves as the most prominent lepidopteran species model system for molecular genetic analysis in lepidopterans (Xu and O’Brochta 2015; Baci *et al.* 2021). It was the first lepidopteran species with a fully sequenced genome, it is the focus of several extensive genomic databases and related resources, and there are robust methods for genetic manipulations (e.g. CRISPR) established in this species (Xu and O'Brochta 2015; Lu *et al.* 2019; Baci *et al.* 2021). Thus, silkworm moths currently present excellent opportunities for pursuing further research into 2 striking noncanonical mechanisms of meiotic chromosomal segregation.

As an initial step toward understanding the differences in meiotic chromosome segregation in male and female silkworms, we set out to create a toolkit that could be used to investigate and compare features in these 3 distinct meiotic processes. In this report, we describe the formation of this toolkit, which includes: (1) establishing a list of proteins that encompass many processes of meiosis (cohesion, SC, recombination), and identifying their orthologs by advanced sequence searches; (2) developing of a new database application to more rapidly identify orthologs; (3) cloning and expressing a number of these genes for antibody production, and analyzing the localization of a subset of the antibodies in the canonical meiosis observed in males by standard immunofluorescence microscopy. In the future, this toolkit will be used to study meiotic structures and processes in both sexes in *B. mori*.

## Methods

### Rearing of silkworms

Silkworm eggs were purchased through https://www.educationalscience.com. Silkworm eggs were hatched in a petri dish at room temperature. After hatching larvae were fed a diet of fresh mulberry leaves collected in the wild and feeding boxes were changed regularly. All tissue samples were obtained from fifth instar larvae, a developmental stage that contains eupyrene spermatocytes and sperm bundles (Cheng *et al.* 2022).

### Ortholog discovery application

A new ortholog discovery application was generated using the OMA (v2.5.0) standalone package (https://omabrowser.org/standalone/), a tool for inferring orthologous genes across species of interest, with default settings to predict orthologs between *B. mori* and model organisms with known well-established meiotic and SC proteins (human, budding yeast, and fly) (Altenhoff *et al.* 2019). For *B. mori*, the protein sequences were downloaded from National Center for Biotechnology Information (NCBI) (release 103). The sequence information used for human, budding yeast, and fly came from the OMA database (December 2021 release). The primary OMA results used in downstream analysis were the pairwise orthologs between *B. mori* and each of the individual model organisms. These pairwise orthologs were used to generate an R Shiny web app (R v4.2.0; Shiny v 1.7.2) that allows users to find the *B. mori* orthologs for a list of genes from these 3 model organisms. This ortholog finder is available for use at https://simrcompbio.shinyapps.io/bombyxmoriorthologfinder/.

### Protein alignment

Protein multiple sequence alignments shown in figures were performed using the algorithm T-coffee, which is embedded in the sequence viewer JalView (http://www.jalview.org) and visualized according to the Clustal coloring scheme (https://www.jalview.org/old/v2_8/help/html/colourSchemes/clustal.html) (Larkin *et al.* 2007). Coiled-coil (CC) regions were identified using DeepCoil from the MPI Bioinformatics Toolkit (https://toolkit.tuebingen.mpg.de/tools/deepcoil) (Ludwiczak *et al.* 2019). Predicted conserved functional domains were identified with NCBI blastp suite (https://blast.ncbi.nlm.nih.gov/Blast.cgi?PAGE=Proteins) or Prosite (https://prosite.expasy.org/).

### Antibody preparations

cDNAs of the silkworm genes were obtained by RT-PCR using the SuperScript III First-Strand Synthesis System (Invitrogen) with RNA extracted from fifth instar testes using TRIzol Reagent (Ambion) and random primers supplied with the kit. PCR was performed on cDNA with gene specific primers for *sycp1* (5′-CGGGATCCGACGCGCATGCTTCCCTCGAGGCATCATCG-3′ and 5′-CCCAAGCTTCAGCACCCGCGCCACGCGCACACACTC-3′), *sycp2* (5′-cgggatccTTGTGTAAGTCTTTGTATGACGCGCACAAA-3′ and 5′-ccgctcgagACTAAAGTCTTCCTTCAGCAATTGTACCAT-3′), *sycp3* (5′-ataagaatGCGGCCGCATGTCTTCAAAGAAATGTGGAAAATCGAAATTTAT-3′ and 5′-cgGGATCCTCAAAAATCATTTTGCATGGCATGAAATATTGTTC-3′), *hop1* (5′-cgcggatcc ATGAAACAGCTGACTGTCATAGCGGTCAG-3′ and 5′-CcgctcgagGCCGCAGGTGAGCAGTGCCGCGTCGTCG-3′), *pch2* (5′-cgggatccATGAATTTAAAAAATTCATTACACGTCGAA-3′ and 5′-ccgctcgagATGTCCATTAGTAACTACTACAGGAGG-3′), *smc1* (5′-cgggatccATGCCTGCGTTTCTTAAATATATCGA-3′ and 5′-ccgctcgagCTTTGCGAGC TCTCTCTGTACGGT-3′), and *smc3* (5′-cgggatccATGCACATAAAACAGGTAATAATCCA-3′ and 5′-ccgctcgagTTTCTCTGATATTTGCTGTCTTAAG-3′) and directly cloned into the pET21a vector (Novagen) using compatible restriction sites within the plasmids and PCR primers for each gene. The cloned genes were expressed in BL21(DES) bacterial cells and purified under denaturing conditions using the in-frame 6XHIS tag in the vector and Ni-agarose beads (MilliporeSigma). Purified proteins including SYCP1 (amino acids 279–512), SYCP2 (amino acids 1,470–1,789), SYCP3 (full-length protein), SMC1 (amino acids 1–280), SMC3 (amino acids 1–340), HOP1 (amino acids 34–296), and PCH2 (full-length protein) were used to generate antibodies at Cocalico Biologicals.

### Immunostaining and microscopy

Primary antibodies were used at 1:250 as follows: anti-rabbit SYCP1, anti-guinea pig or anti-rat SYCP2, anti-guinea pig SYCP3, anti-rat HOP1, anti-rat SMC1, anti-guinea pig SMC3, and anti-rabbit PCH2 (made but did not work in pachytene nuclei from spermatocyte spreads). Secondary goat anti-guinea pig, rabbit, rat, or mouse Alexa-488, Alexa-555, and Alexa-647 IgG H&L chain conjugated antibodies were used at 1:500 (Molecular Probes, Life Technologies).

For preparation of meiotic chromosome spreads from spermatocytes, testes were freshly dissected from fifth instar larvae, washed in PBS, and briefly washed in hypotonic buffer (0.1 M sucrose, 5 mM EDTA, 0.1 mM phenylmethylsulphonyl fluoride in water) 2 times. Testes were transferred to a new tube and incubated in hypotonic buffer for 30 min on ice. Supernatant was replaced with 100 µl of hypotonic buffer, and testes were homogenized with pestle. Spermatocyte suspension was incubated for 30 min on ice. Fixative (1.6% paraformaldehyde, 0.15% Triton-X, 0.05 M sucrose, 5 mM EDTA in water) was applied on slides and 2 µl of spermatocyte suspension was spread onto the slides. Slides were placed in a humidified chamber, fixed for 3 h, then air dried for 30 min at room temperature. Slides were washed with wash buffer (0.1% propylene glycol, 0.04% Triton-X in water) for 20 min 2 times, air dried at room temperature, then stored at −70°C.

Prior to immunostaining, an antigen retrieval step was performed where the slides were put in 10 mM sodium citrate buffer (pH 6.0) at 90°C for 20 min. The slides were allowed to cool in solution before being immersed into PBS + 0.1% Tween (PBST) for 5 min. Slides were incubated with blocking solution (PBST + 0.5% BSA) for at least 2 h before primary antibodies were applied in PBST at 1:250 dilution at 4°C overnight. Slides were washed in PBST before secondary antibody was applied in PBST (1:500 dilution) overnight at 4°C. Slides were again washed before mounting in Vectashield (Vector Laboratories/VWR) containing DAPI. Immunofluorescent images were acquired with a DeltaVision microscopy system (Leica Microsystems) consisting of a 1 × 70 inverted microscope with a high-resolution CCD camera.

For TEM analysis, dissected testes were prefixed with 2.5% paraformaldehyde and 2% glutaraldehyde in 50 mM sodium cacodylate containing 1% sucrose (pH 7.4). The samples were post fixed in 2% OsO4. After dehydration with a graded ethanol series, samples were infiltrated and embedded in Epon resin (EMS, Fort Washington, PA). Ultrathin (60–80 nm) sections were cut with a diamond knife and collected on single-slot copper grids. Grids were poststained with 2% uranyl acetate and 1% lead citrate. Images were acquired on an FEI transmission electron microscope (Tecnai Bio-Twin12, FEI) at 80 kV.

### Yeast two-hybrid

Lysate was prepared from testes by vortexing with acid-washed glass beads (Sigma-Aldrich) in PBS for 10 min. Total RNA was extracted from the lysate using a Maxwell 16 instrument (Promega) and Maxwell RSC simplyRNA Tissue kit (Promega). Five micrograms of total RNA were used for cDNA synthesis using the SuperScript IV First-Strand Synthesis System (ThermoFisher) and Oligo (dT)_20_ primers according to kit protocol. Reactions were treated with RNase H prior to target-specific PCR amplification.

Binding domain pGBDU-C1 (*URA3*) and activation domain pGAD-C1 (*LEU2*) (James *et al.* 1996), were linearized via PCR, and gel purified. Constructs were assembled using NEBuilder HiFi DNA Assembly Master Mix (New England BioLabs) with the exception of *hop1*, which was synthesized as a gBlock (Integrated DNA Technologies) and cloned directly into the vector with compatible restriction sites. Sanger sequencing was performed on all constructs to verify in-frame fusion prior to transformation.

Binding domain constructs were transformed via polyethylene glycol (PEG molecular weight 3,350) (Sigma-Aldrich) and salmon sperm DNA (ThermoFisher) into PJ69-4A, *MAT***a***trp1-901 leu2-3 leu2-112 ura3-52 his3-200 gal4Δ gal80Δ LYS2::GAL1-HIS3 GAL2-ADE2 met2::GAL7-lacZ*, and activating domain constructs into *MAT***a***trp1-901 leu2-3 leu2-112 ura3-52 his3-200 gal4Δ gal80Δ LYS2::GAL1-HIS3 GAL2-ADE2 met2::GAL7-lacZ* (James *et al.* 1996), and transformants selected on synthetic dextrose (SD)-URA or SD-LEU plates, respectively. Strains were mated overnight in rich media, and diploids selected on SD-LEU-URA plates. Individual transformants were grown overnight in SD-LEU-URA liquid media, and equivalent amounts of cells were spotted in 10-fold serial dilutions to SD-LEU-URA and SD-HIS media. Plates were allowed to grow for 3–5 days at 30°C.

## Results and discussion

### Identifying orthologs of known meiotic proteins in *B. mori*

We set out to identify *B. mori* orthologs of genes that function in a diverse set of meiotic processes. These included proteins involved in the condensin and cohesin complexes at the chromosome axis, proteins of the SC at both the lateral element (LE) and central region, regulators of SC formation, meiotic progression/cell cycle, double-strand break (DSB) formation and repair, and proteins required for crossover formation (Table 1). After gathering a preliminary list of such genes, we used sequences from mouse, human, plants, or budding yeast homologs to perform blastp searches of *B. mori* sequences contained in the NCBI nonredundant protein database. This blastp database search process facilitated the discovery of a number of the sought-after orthologs in each of these categories (noted as blastp in Supplementary Table 1).

**Table 1.** Selection of potential meiotic homologs identified in *B. mori*.

Chromosome axis	SC	Meiotic progression/cell cycle	DSB formation	DSB repair	Copromoting proteins
**Condensin**	**Axial/LE**	PCH2*^a^*	SPO11	RAD51	MSH4
SMC2	HOP1*^a^*	CDC7	–	DMC1	MSH5
SMC4	SYCP2*^a^*	POLO	–	RAD54	MLH1
CAP-H	SYCP3*^a^*	AURORA A	–	RAD50	MLH3
CAP-H2	**Central region**	AURORA B	–	MRE11	RNF212
CAP-G	SYCP1*^a^*	MSP1	–	RPA1	HFM1/MER3
CAP-D2	**SC regulator**	HUS1	–	MCM8	NARYA
CAP-D3	SINA	BUBR1	–	BLM1	–
**Cohesin**	–	CDK2	–	NABP2	–
SMC1^a^	–	CDK4	–	MUS81	–
SMC3^a^	–	–	–	RECQ5	–
RAD21	–	–	–	BRAC2	–
SA2	–	–	–	NBS1	–
PDS5	–	–	–	–	–

Gene identifiers can be found in Supplementary Table 1.

Antibody to *B. mori* homolog has been made (this article).

Because this process was time consuming and required single input sequences from the potential homologous protein, we looked for a way to streamline the identification of orthologs. Utilizing our new ortholog finder application (noted as Shinyapp in Supplementary Table 1; see *Methods*), we rapidly identified potential homologs in all categories. For example, the cohesin subunit orthologs, SMC1 and SMC3, were identified using yeast and human proteins, respectively. We then aligned both predicted *B. mori* proteins to the respective mouse sequence and found that *B. mori* predicted SMC1 protein is 51% identical to mouse SMC1 (Supplementary Fig. 1) and the predicted SMC3 protein is 54% identical to mouse SMC3 (Supplementary Fig. 2), suggesting that we have identified the correct protein for each.

### The SC in *B. mori* males

**Fig. 1.**
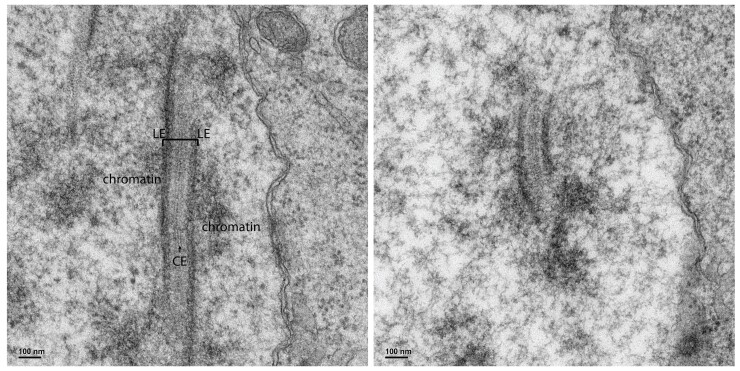
EM images of SC from *B. mori* spermatocytes. Two representative images of the SC in spermatocytes. In the left image, the 2 LEs that attach to the chromatin of the homologous chromosomes are labeled and shown by the bracket. The LEs, which contain axial element components and cohesin complexes, are attached to each other along their length by TF proteins. The overlapping TFs form an electron dense region in the middle called the central element (CE). Scale bar, 100 nm.

*Bombyx mori* males produce eupyrene sperm by canonical meiotic processes. In these processes, the SC is required for the synapsis of homologous chromosomes and plays important roles in pairing and recombination during prophase of meiosis I [reviewed in Zickler and Kleckner (2015)]. Accordingly, the SC is required to ensure proper segregation at the first meiotic division, and alterations or removal of this structure can lead to meiotic failures and aneuploidy (Hassold *et al*. 2007; Geisinger and Benavente 2016; Gao and Colaiácovo 2018; Hughes *et al.* 2018; Kurdzo *et al.* 2018). The SC is a unique structure in that the overall order or pattern is highly conserved between organisms, forming an ∼100 nm evolutionarily conserved tripartite structure that can be seen at high resolution (Moses 1968; Zickler and Kleckner 1999; Cahoon *et al.* 2017). King and Akai in the early 1970s identified that *B. mori* males formed the conventional SC structure (King and Akai 1971; Rasmussen 1976). We verified that structure in males from our *B. mori* stock using electron microscopy (EM) (Fig. 1).

### Identification of the transverse filament protein SYCP1

We set out to identify the major component of the SC, the transverse filament (TF) protein, which spans the width of the 2 chromosome axes. It has long been known that proteins that build the SC are rapidly evolving at both the gene and protein level, often making it challenging to identify orthologs between species (Grishaeva and Bogdanov 2014; Fraune *et al.* 2016; Hemmer and Blumenstiel 2016; Kursel *et al.* 2021). In fact, many of the SC proteins have only small, conserved motifs or have conservations that can only be seen in the protein's size or in secondary structure signatures (Fraune *et al.* 2012; Kursel *et al.* 2021). For these reasons, we had to employ a variety of techniques to find the ortholog of the TF SC protein, SYCP1, in *B. mori*.

**Fig. 2.**
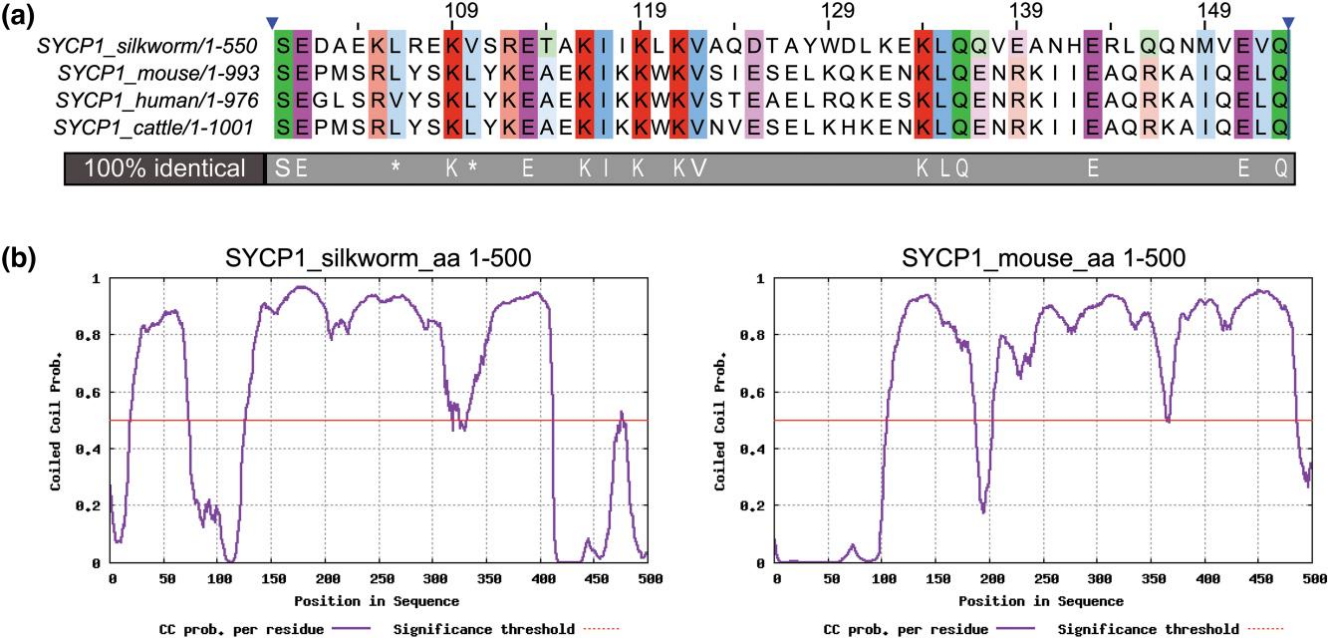
a) The CM1 of SYCP1 in silkworm, mouse, human, and cattle (Fraune *et al.* 2012; Xiang *et al.* 2014). Left blue arrowhead corresponds to aa position 16 for silkworm, aa 96 for mouse, aa 99 for human, and aa 100 for cattle. Full sequence alignment can be found in Supplementary Fig. 4. The conservation threshold for colored shading of the residues was set to 50%. Identical residues are shown below the alignment. (*) indicates residues important for in vitro self-assembly in human SYCP1 (Dunce *et al.* 2018). b) CC prediction of silkworm and mouse SYCP1. Only first 500 aa residues were used for both species in the analysis.

We first used the protein sequences of the TF protein from several organisms, including ZIP1 from yeast and SYCP1 from several mammals, to conduct a blastp search using FLYBASE against the Lepidoptera group containing *B. mori* and *Danaus plexippus* (Monarch butterfly) databases. We identified a hypothetical protein, KGM_212819, with the sequence OWR40882.1 from *D. plexippus*. We used this protein sequence to search in the NCBI database. We obtained a sequence for the SC protein 1 isoform X1 from *Pieris rapae* (Cabbage white butterfly) with sequence ID of XP_022130408.2. We then used this protein sequence to blastp search NCBI *B. mori* database and retrieved the mRNA sequence XM_038019132.1. Primers were designed to this sequence and used to amplify cDNA made from fifth instar *B. mori* testes RNA from our stock. We obtained a sequence that was similar to *B. mori* protein annotated as SOGA3-like transcript (XM_038019132.1). We noted a 360-base insertion within the predicted mRNA that was not found in our cDNA from testes. Analysis of this cDNA sequence (Supplementary Fig. 3) identified a coding region based on a potential start codon in-frame with a stop codon, which was used for further analysis. This coding region was predicted to encode a 550 amino acid protein which is smaller than TF proteins in other well studied organisms [mouse SYCP1 993 aa, human SYCP1 976 aa, budding yeast ZIP1 875 aa, fly C(3)G 744 aa] (Meuwissen *et al.* 1992, 1997; Dong and Roeder 2000; Page and Hawley 2001) suggesting that we may be missing some of the upstream sequence. Analysis of this protein sequence using NCBI blastp identified an SYCP-1 superfamily conserved domain spanning residues 16–310. We then aligned the 550 aa sequence to SYCP1 protein sequences from mouse, human, and cattle (Supplementary Fig. 4). As expected, we observed an overall lack of sequence similarity to other SYCP1 orthologs, however, we noticed that a region which appeared to contain the most similarity was in the position of the conserved motif 1 (CM1) that was identified by Fraune *et al.* (2012) as one of the 2 motifs that had the highest conservation among SYCP1 homologs in vertebrates. CM1 was also one of the regions that allowed Xiang *et al.* (2014) to identify the SYCP1 in *Schmidtea mediterranea* (flatworm). The conservation within the CM1 region for *B. mori* is shown in Fig. 2a.

In addition to the conservation in CM1 for SYCP1, we scanned the protein coding region for CC domains which are common to both TF proteins and proteins of the SC (Page and Hawley 2004). Using DeepCoil Toolkit, we analyzed the first 500 aa of both the predicted *B. mori* protein and that of mice and compared the two. We found that the pattern and length of the CC domain(s) are very similar between the 2 proteins (Fig. 2b). Taken together our results indicate that we have identified all or part of the *B. mori* SYCP1 TF protein.

### Identification of the LE component SYCP2

**Fig. 3.**
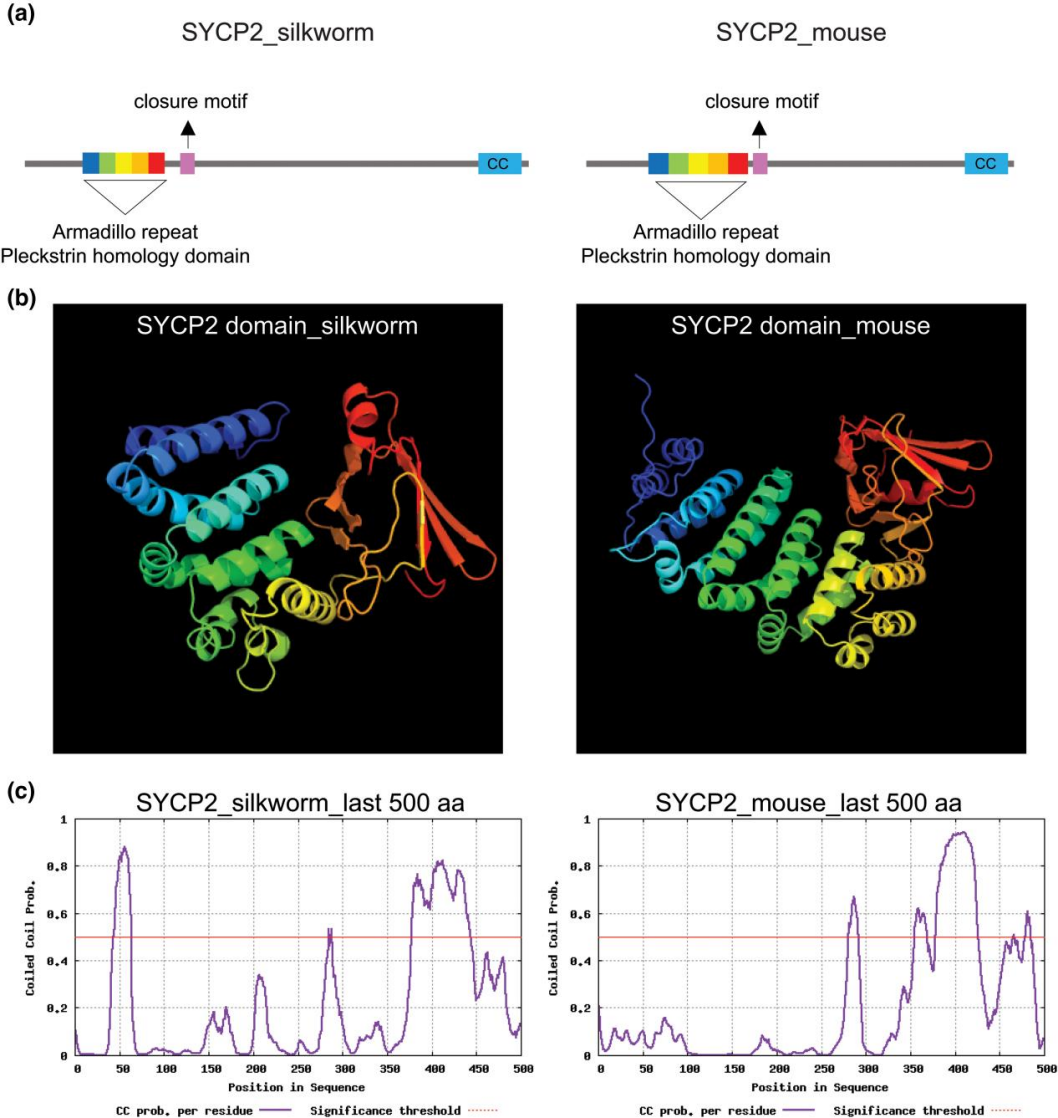
a) Schematic depiction of SYCP2 from silkworm (1,800 aa) and mouse (1,500 aa) showing the location of the Armadillo repeat (rainbow) and the pleckstrin homology domain that make up the SYCP2 domain, the closure motif (pink), and the CC region (turquoise) [reviewed in Ur and Corbett (2021)]. The sequence of these domains for silkworm is shown in Supplementary Fig. 5. b) The secondary structure prediction of the SYCP2 domain in silkworm and mouse. The secondary structure of SYCP2 from silkworm and mouse was generated using Phyre2 web portal (http://www.sbg.bio.ic.ac.uk/∼phyre2/html/page.cgi?id=index). For silkworm SYCP2, Phyre2 identified with 98.1% confidence the protein database molecule SYCP2 domain of 295 residues, and silkworm SYCP2 identified a SYCP2 domain of 371 residues with 100% confidence [shown in rainbow colors which correspond to the same colors in the schematic in (a)]. c) CC prediction of silkworm and mouse SYCP2. The last 500 aa residues were used for both species in the analysis. The location of the CC domain is shown in the schematic in (a).

We next set out to identify SYCP2, a major component of the axial/LE which plays a vital role in the assembly of the SC. When we did not obtain any obvious homologs using standard blastp searches with mammalian SYCP2 sequences, we attempted the search again with mouse SYCP2 using the iterative similarity search tool psi-blast (https://www.ebi.ac.uk/Tools/sss/psiblast/) which utilizes position-specific scoring matrices. We obtained several potential insect SYCP2-like proteins and used these sequences to search the NCBI *B. mori* database. We identified 2 isoforms (isoform X2 XP_037874110.1 and X1 XP_037874109.1) of an uncharacterized PFB0145c-like protein. These 2 isoforms differed by 189 aa at the N-terminus making the smaller of the isoforms 1,611 aa and the longer isoform 1,800 aa. Upon further analysis of the secondary structure of these proteins using the Phyre2 web portal, the longer isoform contained the entire SYCP2 domain, consisting of an Armadillo repeat and a pleckstrin homology domain, found within other SYCP2 homologs, suggesting that the longer isoform is the full-length protein (Fig. 3, a and b; Feng *et al.* 2017; Tromer *et al.* 2021; reviewed in Ur and Corbett 2021). Just downstream from the SYCP2 domain, we identified a closure motif (Fig. 3a; Supplementary Fig. 5) which in other organisms is necessary for SYCP2's interaction and recruitment of HORMA domain containing proteins to the chromosome axes (West *et al.* 2019; reviewed in Ur and Corbett 2021).

We next evaluated the C-terminal region of the predicted *B. mori* SYCP2 1,800 aa protein for a CC domain which has been shown to be evolutionarily conserved in SYCP2 proteins (Yang *et al.* 2006; Tromer *et al.* 2019) and necessary for the interaction with SYCP3, a component of the SC axes (West *et al.* 2019). We found that our predicted *B. mori* SYCP2 did indeed have a predicted CC domain of similar size in the C-terminal region of the protein suggesting we found the SYCP2 homolog (Fig. 3, a and c).

### Identification of orthologs for SYCP3, HOP1, and PCH2

We also aimed to identify and evaluate potential orthologs of other axial/LE SC components. We were able to rapidly identify SYCP3, a structural component of the SC, and HOP1, an axial element component that is required for homologous chromosome pairing.

**Fig. 4.**
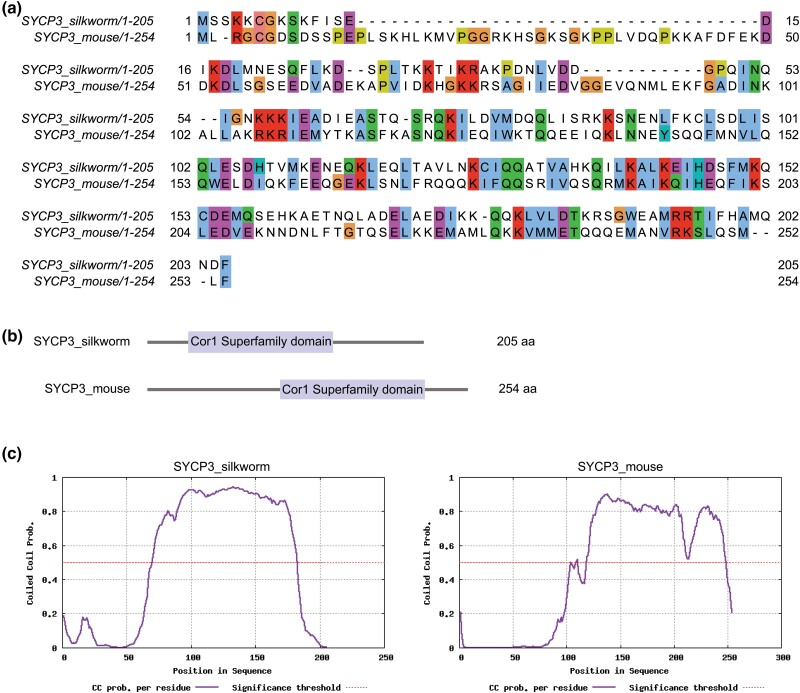
a) Protein sequence alignment of SYCP3 from silkworm and mouse. Default of 30% was used for colored shading of the conservation for residues in JalView. Comparison of silkworm and mouse SYCP3 proteins show 25% identity and 51% positive substitutions. b) Schematic depiction of the location of the Cor1 superfamily domain in SYCP3 of silkworm and mouse. Cor1 superfamily domain for each protein was identified by NCBI blastp search. Cor1 domain in silkworm spans residues 56–170 and in mouse 101–224. c) *Bombyx mori* SYCP3 has a predicted CC domain.

A SYCP3 ortholog was found using a blastp search with the mouse SYCP3 against the *B. mori* database. We identified an uncharacterized protein LOC101742370 (XP_004932505) of 205 aa and evaluated it further. Alignment of the predicted SYCP3 from *B. mori* and mouse SYCP3 (Fig. 4a) showed the most conserved regions overlapped the 2 conserved motifs (CM1 and CM2) identified by Fraune *et al*. (2012) for SYCP3 orthologs. The conserved domain database tool used during the blastp search also identified that, like mouse SYCP3, *B. mori* SYCP3 contained a Cor1 superfamily domain. The Cor1 domain was first identified by Peter Moens’ laboratory in 1994 when he found COR1 which is now known as the ortholog of SYCP3 in hamsters (SYP3) (Dobson *et al.* 1994). The location of the Cor1 domain is shown as a schematic in Fig. 4b.

It was previously shown that CM1 and CM2 found in SYCP3 orthologs flank a CC domain, and therefore we assessed whether the predicted SYCP3 of *B. mori* contained a CC domain. We identified a large, CC domain of similar size and location in *B. mori* SYCP3 compared to mouse SYCP3. This CC region has been shown to be important for the tetrameric assembly of SYCP3 fibers (Syrjänen *et al.* 2014). These collective studies indicate we have identified SYCP3 in *B. mori*.

**Fig. 5.**
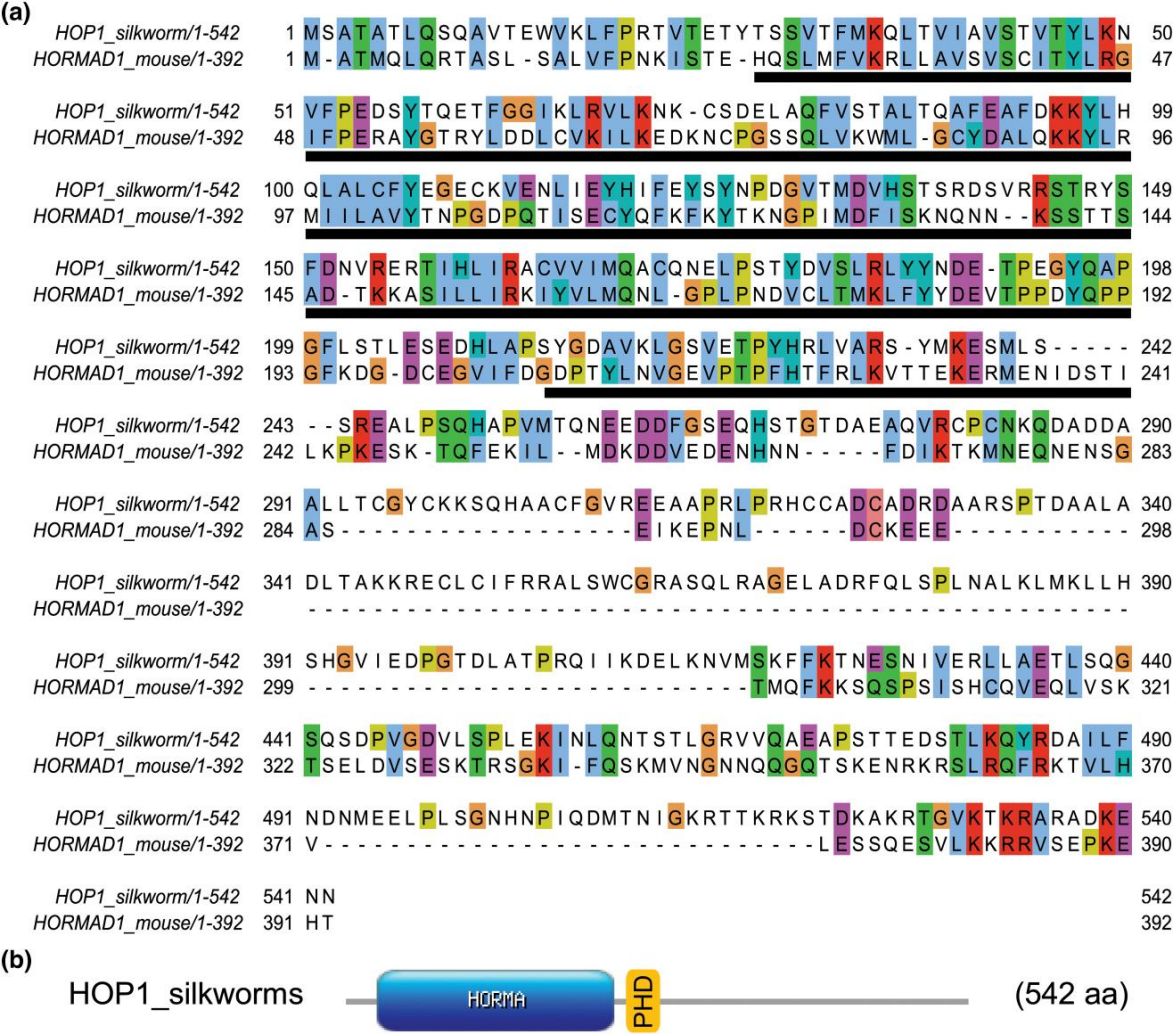
a) Protein sequence alignment of HOP1 from silkworm and HORMAD1 mouse. The HORMA domain sequence is underlined in black. Comparison of silkworm and mouse proteins show 31% identity and 51% positive substitutions. Default of 30% was used for colored shading of the conservation for residues in JalView. b) Schematic depiction of the location of the HORMA domain which spans residues 28–234. The PHD superfamily domain identified by NCBI is just downstream of the HORMA domain at residues 279–325 (orange box).

**Fig. 6.**
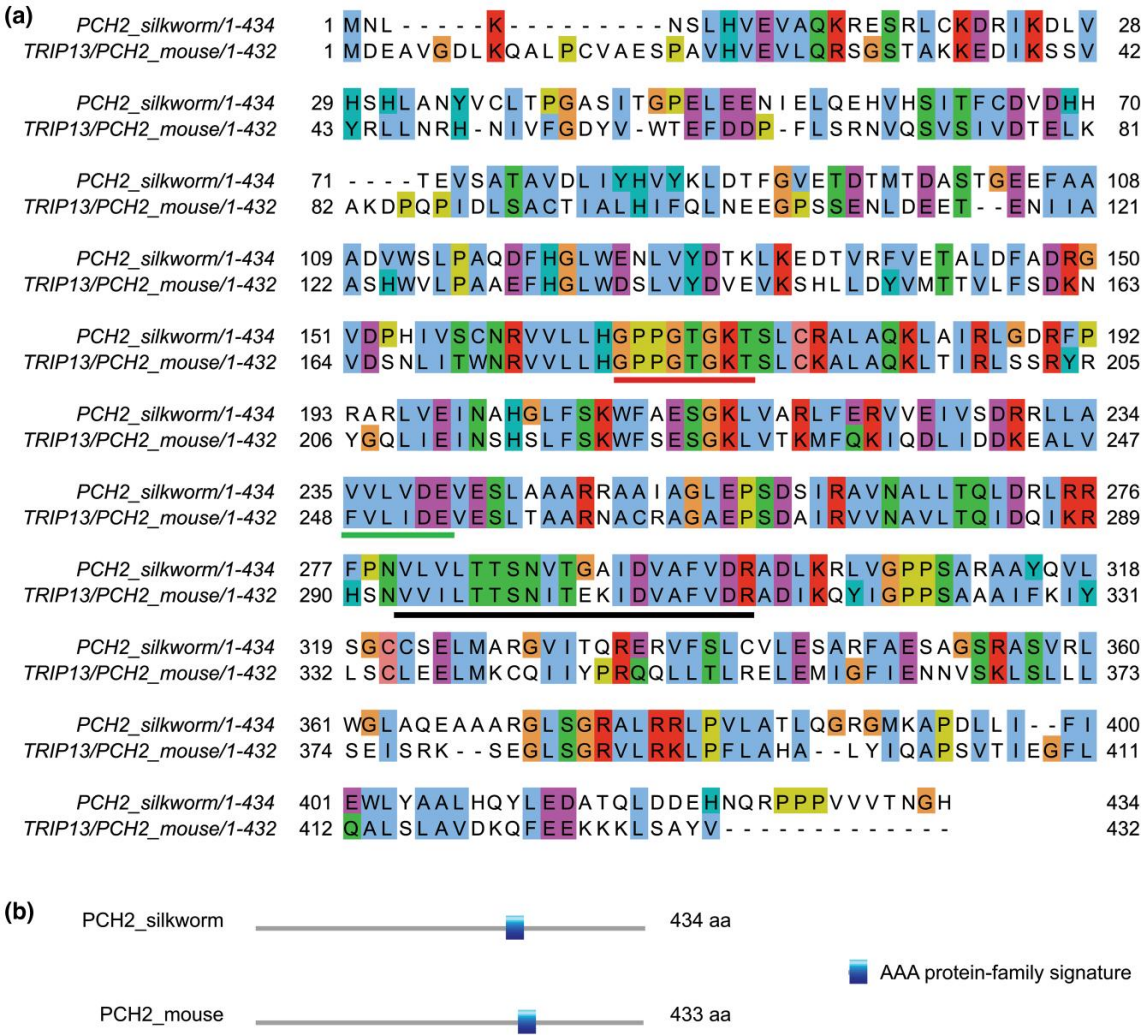
a) Protein sequence alignment of PCH2 from silkworm and mouse. Comparison of PCH2 from silkworm and TRIP13/PCH2 from mouse show 43% identity and 65% positive substitutions. Default of 30% was used for colored shading of the conservation for residues in JalView. Black bar under the sequence is the AAA protein-family signature domain that is a characteristic of AAA + ATPases. Red and green bars under sequence are the canonical Walker A and B motifs, respectively. b) Schematic depiction of the location of the AAA protein-family signature domain and spans residues 280–299 in silkworm and 292–312 in mouse.

We searched for 2 additional highly conserved meiosis-specific proteins (HOP1 and PCH2) that function together in mediating the meiotic prophase checkpoint. To identify HOP1 and PCH2 we searched the NCBI *B. mori* database using the mouse homolog for each protein (HORMAD1 and TRIP13/PCH2). We identified a silkworm ortholog of HOP1 as an uncharacterized 542 aa protein LOC101737689 isoform X1 (XP_021204681) (Tromer *et al.* 2019) and a potential 434 aa PCH2 ortholog as an AAA family ATPase protein (NP_001040375.1). We named the potential *B. mori* homolog of mouse HORMAD1 as HOP1 because the protein is predicted to have a PHD domain common to fungal HOP1 and plant ASY1 by the NCBI blastp search (Ur and Corbett 2021). Sequence comparison of silkworm and mouse HOP1 identified the strongest conservation in the region of the protein containing the HORMA domain (Fig. 5, a and b). Sequence comparison of silkworm PCH2 and mouse TRIP13/PCH2 showed an overall 43% sequence identity. Prosite scan identified an AAA-protein family signature that is found in other PCH2 orthologs, and sequence comparison with PCH2 orthologs identified canonical Walker A and B motifs (Fig. 6, a and b; Chen *et al.* 2014).

### Verification of identified proteins as orthologs

**Fig. 7.**
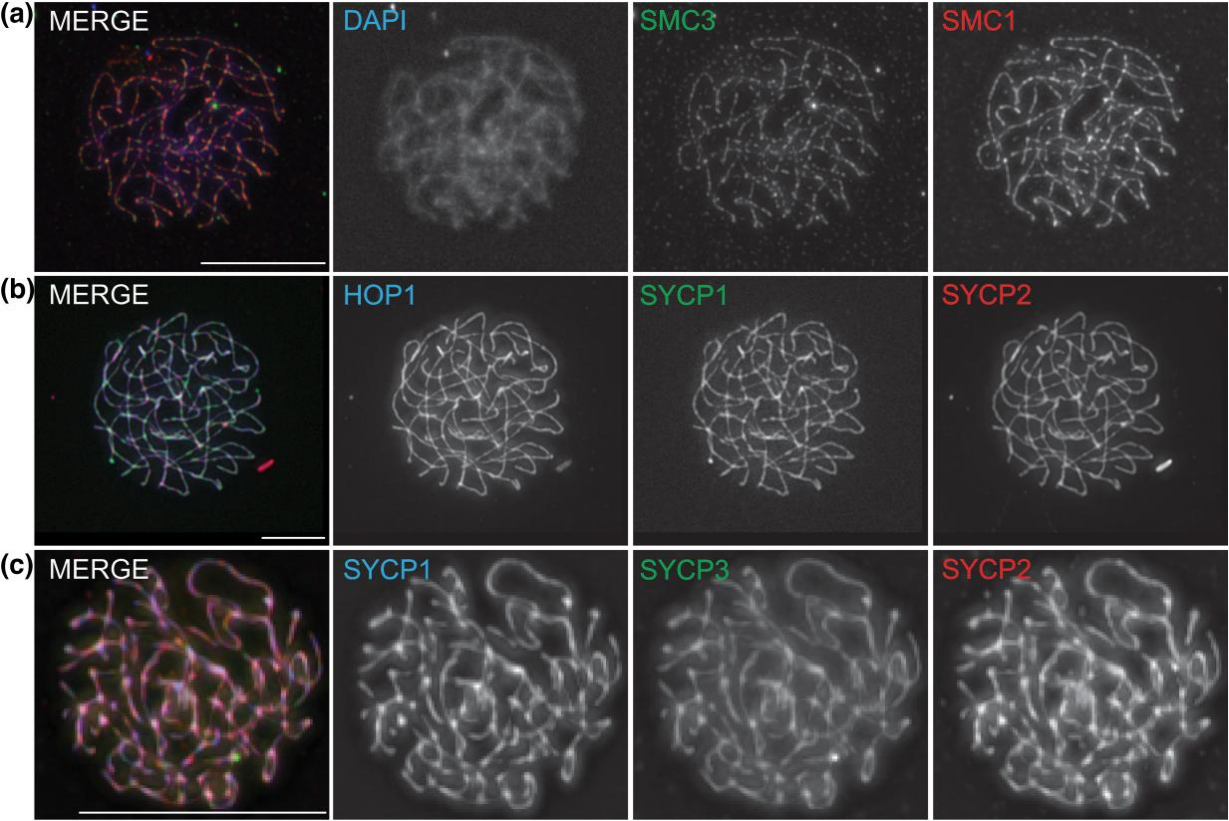
Meiotic chromosome spermatocyte spreads of pachytene nuclei. a) Immunostaining of pachytene nuclei stained with DAPI (blue), SMC3 (green), and SMC1 (red) in the merge with individual panels shown in white. Cohesin complex containing SMC1/3 localizes along the chromatin axes. b) Immunostaining of pachytene nuclei stained with HOP1 (blue), SYCP1 (green), and SYCP2 (red) in the merge with individual panels shown in white. Axial/LE components HOP1 and SYCP2 coassociate with the SC TF component SYCP1. c) Immunostaining of pachytene nuclei stained with SYCP1 (blue), SYCP3 (green), and SYCP2 (red) in the merge with individual panels shown in white. SYCP2 and SYCP3 both coassociate with SYCP1 on the SC. All image panels are projections. Scale bars, 15 μm.

We generated polyclonal antibodies against all or part of the protein sequence for SMC1, SMC3, SYCP1, SYCP2, SYCP3, HOP1, and PCH2 (see *Methods*) and assayed their localization in spermatocyte spreads of *B. mori* during pachytene. In all cases (except PCH2 which did not work well in pachytene spermatocytes), using the antibodies to the selected proteins we observed a well-established localization pattern. Figure 7a shows the localization of cohesin (SMC1 and SMC3) along the chromatin axes at the lateral sides of the SC where it mediates cohesion of sister chromatids. In pachytene, and at this resolution, cohesin staining pattern resembles that of the SC. Figure 7, b and c shows the SC structure using antibodies to the TF protein SYCP1 and the coassociation of the axial/LE proteins SYCP2, SYCP3, and HOP1. At this resolution, these proteins colocalize and show the typical full-length SC structure found between paired homologs at the pachytene stage of meiosis.

**Fig. 8.**
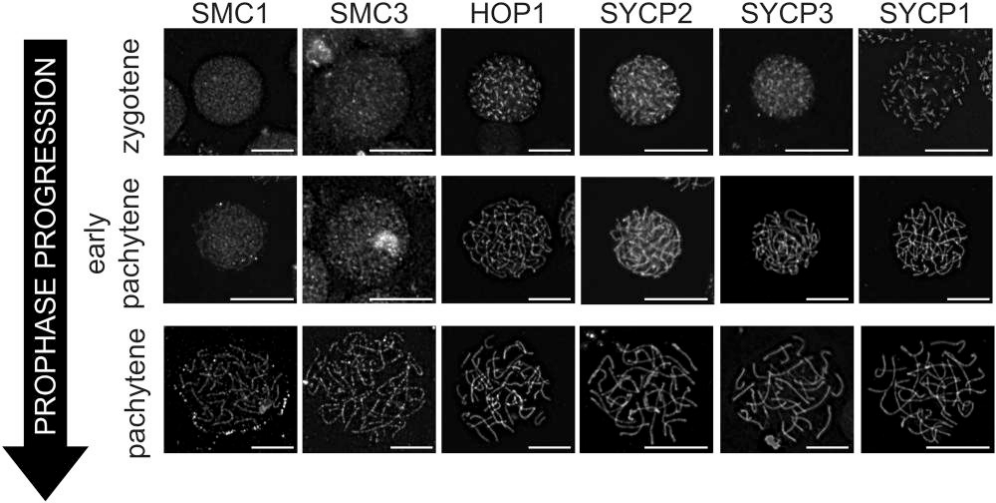
Meiotic chromosome spermatocyte spreads of zygotene, pachytene, and late pachytene nuclei. Staging of nuclei was determined based on SC morphology within each nucleus and shown is only the denoted antibody (SMC1, SMC3, HOP1, SYCP2, SYCP3, and SYCP1) for each image. Scale bars, 10 μm.

Once we identified the SC structure, we further analyzed meiotic progression in our fifth instar spermatocyte spreads. Using SC maturation as an indicator of meiotic staging, we were able to identify nuclei at various stages of meiotic prophase for all antibodies tested. Figure 8 shows spermatocyte nuclei from zygotene throughout pachytene. Precise staging will only be able to be determine when markers for additional meiotic events (DSB formation/repair and crossover progression) become available. It is important to note that while exact staging cannot be determined currently with the markers available, *B. mori* SC formation does follow general trends consistent with other organisms in that axial element/LE components we identified, for instance SYCP2/SYCP3, appear to load prior to full synapsis, as would be indicated by SYCP1 thread like staining (Gray and Cohen 2016). Consistent with similar SC formation trends, antibody specificity was apparent in the spermatocyte spreads. In addition to seeing various stages of meiotic prophase, we often observed nuclei that lacked markers of the SC but contained diffuse SMC1/3 staining (Supplementary Fig. 6). These nuclei likely represent spermatogonia or very early primary spermatocytes in leptotene.

**Fig. 9.**
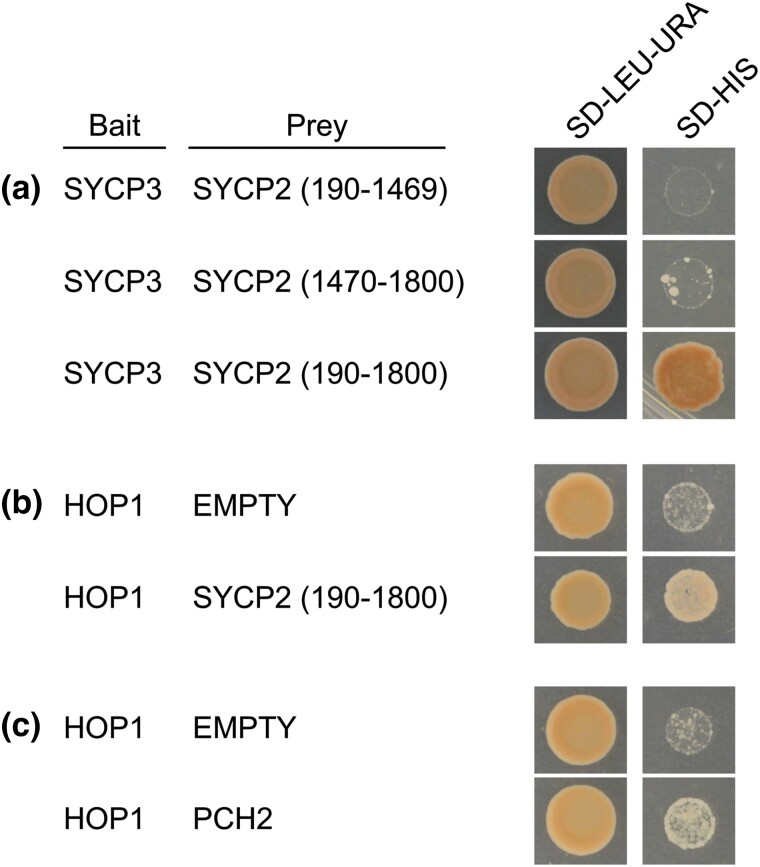
Interaction of *B. mori* proteins using yeast two-hybrid assay. a) Only residues 190–1,800 of SYCP2 interacts with SYCP3 on the selection plate SD-HIS. SYCP2 N-terminal and C-terminal residues were 190–1,469 and 1,470–1,800, respectively. Note that when full length SYCP2 (1–1,800) was tested, no interaction was found. This could be due to structural constraints of the SYCP2 domain near the GAL4-AD of the vector or unknown constraints using the yeast two-hybrid systems. b) HOP1 and SYCP2, as well as (c) HOP1 and PCH2 interact based on growth on the selection plate SD-HIS. HOP1 does not interact with empty prey vector [shown in (b) and (c) for independent experiments].

In addition, we performed protein–protein interaction studies using a yeast two-hybrid assay to look for known interactions between the axial/LE components. While we did not find an interaction using full-length SYCP2, an N-terminal truncation which removes part of the SYCP2 domain (190–1,800 aa), was able to interact with SYCP3. This N-terminal deletion is the predicted sequence of isoform X2 (Supplementary Fig. 5) and removes part of the SYCP2 domain of the full-length protein. The SYCP2–SYCP3 interaction required most of the SYCP2 protein sequence, as no interaction was seen when this fragment was tested in 2 parts (Fig. 9a). In addition, we identified a protein–protein interaction for SYCP2–HOP1 (Fig. 9b), and the known interaction of HOP1–PCH2 using the potential PCH2 ortholog identified in our search (Chen *et al.* 2014; Fig. 9c). The latter interaction and the fact that our PCH2 candidate was expressed in testes suggests that we have identified a potential PCH2 ortholog worth further analyzing, as well as those for SYCP2 and HOP1.

## Conclusions

While the canonical mechanisms of meiosis and crossing over are very similar in most sexually reproducing species, there are also a notable number of taxa in which alternative mechanisms have evolved to facilitate meiotic chromosomal segregation without recombination, at least in 1 sex (Wolf 1994; Zickler and Kleckner 1999). *Bombyx mori* is a classic example of the meiotic diversity seen in Lepidoptera in that females’ complete meiosis in the absence of recombination, while males have both a canonical chiasmate meiosis and undergo an atypical meiosis where the product is anucleated sperm. Understanding how females segregate homologs accurately in the absence of crossing over, as well as how meiotic proteins behave during the formation of apyrene sperm in males, will greatly increase our knowledge of sexual reproduction in this large and prominent insect order.

In both *Drosophila* males and Lepidoptera females, the heterogametic sex utilizes protein aggregates to ensure accurate segregation of their chromosomes during meiosis. In the case of *Drosophila* males, proteins are used to create pseudo-chiasmata on each chromosome pair (Cooper 1964; Kabakci, Reichle, *et al.* 2022; Kabakci, Yamada, *et al*. 2022; Vernizzi and Lehner 2022). In Lepidoptera females, segregation of chromosomes relies on a large proteinaceous structure that forms between the homologs, which is thought both to prevent crossing over and to ensure accurate chromosome segregation (Rasmussen 1976, 1977a, 1977b; Wolf 1994). Since the canonical structure of the SC was seen in early meiosis I in *B. mori* females, it was speculated that this large protein-dense structure was a remodeling of canonical SC and was given the name modified SC (MSC). Currently, little is known concerning the molecular structure, components, or function of the MSC. Indeed, whether the structure is in fact composed of canonical SC proteins remains a fundamental outstanding question.

Using a variety of techniques, we were able to identify proteins that play a role at the chromosome axes (condensin and cohesin), in the SC, in the formation or repair of double-strand breaks, which are necessary for crossing over, and those that could be involved in meiotic checkpoints. We have identified and further characterized the TF component of the SC SYCP1, the axial/LE components SYCP2, SYCP3, and HOP1, and the meiotic checkpoint protein PCH2. Using immunolocalization or protein–protein interaction studies, we were able to verify that they are indeed the true orthologs in *B. mori*. These reagents provide us with the toolkit necessary to begin the analysis of the structure known as the MSC that forms between homologs during meiosis I in lepidopteran females. It will also advance our understanding of the molecular mechanisms underlying the “Haldane-Huxley” rule of repeatedly evolving achiasmy in the heterogametic sex (Burt *et al.* 1991; Hedrick 2007).

## Supplementary Material

jkad058_Supplementary_Data

## Data Availability

Original data underlying this article can be accessed from the Stowers Original Data Repository at http://www.stowers.org/research/publications/libpb-2366. The *Bombyx mori* ortholog finder is available for use at https://simrcompbio.shinyapps.io/bombyxmoriorthologfinder/. Supplemental material is available at G3 online.
