# Supplementary material for: A molecular cell biology toolkit for the study of meiosis in the silkworm Bombyx mori
Source: G3 (Bethesda). 2023 Mar 13;13(5):jkad058. doi: 10.1093/g3journal/jkad058 (PMC10151401; doi:10.1093/g3journal/jkad058)
Supplement: jkad058_Supplementary_Data [file jkad058_supplementary_data.zip › Figure_S1_G3-2023-404089.pdf]

```
SMC1_silkworm/1-1220     1 MPAFLKYIDMENFKTYRGHHRIGPLKSFTAVVGPNGSGKSNFMDAVSFVM   50
   SMC1_mouse/1-1233     1 M-GFLKLIEIENFKSYKGRQIIGPFQRFTAIIGPNGSGKSNLMDAISFVL   49

SMC1_silkworm/1-1220    51 GEKTSLLRVKRLSDLIHGASINKPVSRSASVTATFVLEDMTEKHFQRSVI  100
   SMC1_mouse/1-1233    50 GEKTSNLRVKTLRDLIHGAPVGKPAANRAFVSMVYSEEGAEDRTFARVIV   99

SMC1_silkworm/1-1220   101 GQSSEHKIDGQSVSVSNYLGELEKLGINVKAKNFLVFQGAVESIAMKNPK  150
   SMC1_mouse/1-1233   100 GGSSEYKINNKVVQLHEYSEELEKLGILIKARNFLVFQGAVESIAMKNPK  149

SMC1_silkworm/1-1220   151 ERTTLFEEISGSGVLKEQYEACRAEVNRADEEAQFSYQKKKGVAAERKEA  200
   SMC1_mouse/1-1233   150 ERTALFEEISRSGELAQEYDKRKKEMVKAEEDTQFNYHRKKNIAAERKEA  199

SMC1_silkworm/1-1220   201 KFEKEEAEKYTRLKQELQEQKVELQLFHLYHNEREIQAYEEDLQHKQQEL  250
   SMC1_mouse/1-1233   200 KQEKEEADRYQRLKDEVVRAQVQLQLFKLYHNEVEIEKLNKELASKNKEI  249

SMC1_silkworm/1-1220   251 AKIEKKRQKAEEALKEKKKEAGTVQRELAKIEQDIREVEAEISKKRPTFI  300
   SMC1_mouse/1-1233   250 EKDKKRMDKVEDELKEKKKELGKMMREQQQIEKEIKEKDSELNQKRPQYI  299

SMC1_silkworm/1-1220   301 KAKERVTHTQKKLESAQKTLEQARKAHEAHQDDIRTLEEELRTLEQQKAT  350
   SMC1_mouse/1-1233   300 KAKENTSHKIKKLEAAKKSLQNAQKHYKKRKGDMDELEKEMLSVEKARQE  349

SMC1_silkworm/1-1220   351 WETA-SGTGHSGKADVHLEEAQIREYEELKMEASRQAARYLQELDSVNRE  399
   SMC1_mouse/1-1233   350 FEERMEEESQSQGRDLTLEENQVKKYHRLKEEASKRAATLAQELEKFNRD  399

SMC1_silkworm/1-1220   400 QKADQDRLDNELRKKGELENKHRQKGHERNEAVKRVDKLNEHIKSSEQAL  449
   SMC1_mouse/1-1233   400 QKADQDRLDLEERKKVETEAKIKQKLREIEENQKRIEKLEEYITTSKQSL  449

SMC1_silkworm/1-1220   450 EEQRRLRAELQADVGSCRGRAASLQTQLEDVAAQLGDARVDKHEEARRRK  499
   SMC1_mouse/1-1233   450 EEQKKLEGELTEEVEMAKRRIDEINKELNQVMEQLGDARIDRQESSRQQR  499

SMC1_silkworm/1-1220   500 KQEIVESFKRDIPG-VYDRMINMCQPTHKRYNVAITKVLGKYMEAIVVDT  548
   SMC1_mouse/1-1233   500 KAEIMESIKRLYPGSVYGRLIDLCQPTQKKYQIAVTKVLGKNMDAIIVDS  549

SMC1_silkworm/1-1220   549 EKTARRCIQVLKERMLEPETFLPLDYIQAKPLRERLRDIKEPKNVKLLFD  598
   SMC1_mouse/1-1233   550 EKTGRDCIQYIKEQRGEPETFLPLDYLEVKPTDEKLRELK---GAKLVID  596

SMC1_silkworm/1-1220   599 VLRFEPAAIHRAVLFVTNNALVCETPEDASRVAYDLDRNKNSRYDALALD  648
   SMC1_mouse/1-1233   597 VIRYEPPHIKKALQYACGNALVCDNVEDARRIAFG----GHQRHKTVALD  642

SMC1_silkworm/1-1220   649 GTFYQKSGIISGGSLDLARKAKRWDEKHLSQLKAKKEKLTEELRESMKKS  698
   SMC1_mouse/1-1233   643 GTLFQKSGVISGGASDLKAKARRWDEKAVDKLKEKKERLTEELKEQMKAK  692

SMC1_silkworm/1-1220   699 RKESELTTVDSQIRGLESRLKYAVTDRDTTLKQIKTLD-AEIVELERKME  747
   SMC1_mouse/1-1233   693 RKEAELRQVQSQAHGLQMRLKYSQSDLEQTKTRHLALNLQEKSKLESELA  742

SMC1_silkworm/1-1220   748 TFGPQIEEIERTIRLRDAKIQEVKENMNNVEDVVFKAFCRDIGVANIRQY  797
   SMC1_mouse/1-1233   743 NFGPRINDIKRIIQSREREMKDLKEKMNQVEDEVFEEFCREIGVRNIREF  792

SMC1_silkworm/1-1220   798 EERELRAQQERAKRRMEFEAQIDRVASNLEFERS--RDTQKNVTRWERAV  845
   SMC1_mouse/1-1233   793 EEEKVKRQNEIAKKRLEFENQKTRLGIQLDFEKNQLKEDQDKVHMWEQTV  842

SMC1_silkworm/1-1220   846 QDAEDELEGGRQAEAKQRADIDHELRRADTLKADRAAARTHLEKAEEDVN  895
   SMC1_mouse/1-1233   843 KKDENEIEKLKKEEQRHMKIIDETMAQLQDLKNQHLAKKSEVNDKNHEME  892

SMC1_silkworm/1-1220   896 KARKEVSSIQKDIQSVQKQMASIEARIESKRSERHNILRQCKIDDIIIPL  945
   SMC1_mouse/1-1233   893 EIRKKLGGANKEMTHLQKEVTAIETKLEQKRSDRHNLLQACKMQDIKLPL  942

SMC1_silkworm/1-1220   946 LEGSLDDTADTES-------DPSSMSTTQQYRKESRIRVDYSMLSDSLRD  988
   SMC1_mouse/1-1233   943 SKGTMDDISQEEGSSQGEESVSGSQRTSSIYAREALIEIDYGDLCEDLKD  992

SMC1_silkworm/1-1220   989 LEEADEVRRRADKLQKAINSLQTTVDKIQAPNMRAMQKLTEVREKVNATN 1038
   SMC1_mouse/1-1233   993 AQAEEEIKQEMNTLQQKLNEQQSVLQRIAAPNMKAMEKLESVRDKFQETS 1042

SMC1_silkworm/1-1220  1039 EAFVAARKRAHRAKLAFEKVKKERHDKFMDCFEHVANEIDAIYKALAMNQ 1088
   SMC1_mouse/1-1233  1043 DEFEAARKRAKKAKQAFEQIKKERFDRFNACFESVATNIDEIYKALSRNS 1092

SMC1_silkworm/1-1220  1089 SAQAFLGPENPEEPYLDGINYNCVAPGKRFQPMSNLSGGEKTVAALALLF 1138
   SMC1_mouse/1-1233  1093 SAQAFLGPENPEEPYLDGINYNCVAPGKRFRPMDNLSGGEKTVAALALLF 1142

SMC1_silkworm/1-1220  1139 AIHSYQPAPFFLLDEIDAALDNTNIGKVASYIRSKKGC-LQTIVISLKEE 1187
   SMC1_mouse/1-1233  1143 AIHSYKPAPFFVLDEIDAALDNTNIGKVANYIKEQSTCNFQAIVISLKEE 1192

SMC1_silkworm/1-1220  1188 FYGCADALVGICSEPADCLVSDVITLSLENYAD--------          1220
   SMC1_mouse/1-1233  1193 FYTKAESLIGVYPEQGDCVISKVLTFDLTKYPDANPNPNEQ          1233
```
