# Supplementary material for: A molecular cell biology toolkit for the study of meiosis in the silkworm Bombyx mori
Source: G3 (Bethesda). 2023 Mar 13;13(5):jkad058. doi: 10.1093/g3journal/jkad058 (PMC10151401; doi:10.1093/g3journal/jkad058)
Supplement: jkad058_Supplementary_Data [file jkad058_supplementary_data.zip › Figure_S2_G3-2023-404089.pdf]

```
SMC3_silkworm/1-1203     1 MHIKQVIIQGFKSYREQIVVEPFDKRHNVVVGRNGSGKSNFFHAIQFVLS   50
   SMC3_mouse/1-1217     1 MYIKQVIIQGFRSYRDQTIVDPFSSKHNVIVGRNGSGKSNFFYAIQFVLS   50

SMC3_silkworm/1-1203    51 DEFSHLRPEQRLALLHEGTGPRVISAFVEIIFDNSDNRIPIEKDEIFLRR  100
   SMC3_mouse/1-1217    51 DEFSHLRPEQRLALLHEGTGPRVISAFVEIIFDNSDNRLPIDKEEVSLRR  100

SMC3_silkworm/1-1203   101 VIGSKKDQFFLNKKVVPRSEVLNLLESAGLSNSNPYYIVKQGKINQMAIA  150
   SMC3_mouse/1-1217   101 VIGAKKDQYFLDKKMVTKNDVMNLLESAGFSRSNPYYIVKQGKINQMATA  150

SMC3_silkworm/1-1203   151 PDSHRLKLLREVAGTRVYDERREESVAILKETVGKVEKINEFLQTIEERL  200
   SMC3_mouse/1-1217   151 PDSQRLKLLREVAGTRVYDERKEESISLMKETEGKREKINELLKYIEERL  200

SMC3_silkworm/1-1203   201 KTLEEEKEELKEYQKWDRARRVLEFIIHDTEHKENKRKLEELEKLRSNSG  250
   SMC3_mouse/1-1217   201 HTLEEEKEELAQYQKWDKMRRALEYTIYNQELNETRAKLDELSAKRETSG  250

SMC3_silkworm/1-1203   251 KEQQHYADLVREAQEHVREANRKLKEARKDVAAAREEKDILSTEQQQLLK  300
   SMC3_mouse/1-1217   251 EKSRQLRDAQQDARDKMEDIERQVRELKTKISAMKEEKEQLSAERQEQIK  300

SMC3_silkworm/1-1203   301 EKTKLELAIKDLTDDVDGDNKSKERAEAELERLRQQISEKERELEELKPK  350
   SMC3_mouse/1-1217   301 QRTKLELKAKDLQDELAGNSEQRKRLLKERQKLLEKIEEKQKELAETEPK  350

SMC3_silkworm/1-1203   351 YEEMKAREEECTRALSLNQQKRQELYAKQGRGTQFTSKQDRDRWIEKELK  400
   SMC3_mouse/1-1217   351 FNSVKEKEERGIARLAQATQERTDLYAKQGRGSQFTSKEERDKWIKKELK  400

SMC3_silkworm/1-1203   401 SLNKQLKDKKDHESKLREDLR-RDANKLTELEKRIEETTKEMERQRVAID  449
   SMC3_mouse/1-1217   401 SLDQAINDKKRQIAAIHKDLEDTEANKEKNLEQY-NKLDQDLNEVKARVE  449

SMC3_silkworm/1-1203   450 EHNKQYYECKKKKDQEQSARNELWRKETSLTQNLSSLKDDLAKADQALRS  499
   SMC3_mouse/1-1217   450 ELDRKYYEVKNKKDELQSERNYLWREENAEQQALAAKREDLEKKQQLLRA  499

SMC3_silkworm/1-1203   500 MAGKPILNGRDSVRKVLETFQERGGDWAKIATQYYGPVIENFTCDKTIYT  549
   SMC3_mouse/1-1217   500 ATGKAILNGIDSINKVLEHFRRKGIN-QHVQNGYHGIVMNNFECEPAFYT  548

SMC3_silkworm/1-1203   550 AVEVTAGNRLFHHIVESDTVGTKILKEMNRQNLPGEVTFMPLNRLQVRDM  599
   SMC3_mouse/1-1217   549 CVEVTAGNRLFYHIVDSDEVSTKILMEFNKMNLPGEVTFLPLNKLDVRDT  598

SMC3_silkworm/1-1203   600 VYPNDNNAIAMVQKLKYDPKYAKAMKYIFGKTLICRNLECATELGKQFHL  649
   SMC3_mouse/1-1217   599 AYPETNDAIPMISKLRYNPRFDKAFKHVFGKTLICRSMEVSTQLARAFTM  648

SMC3_silkworm/1-1203   650 DCVTLEGDQVSSKGSLTGGYFNQSRSRLEMQKTRSELMEQITTLDLELS-  698
   SMC3_mouse/1-1217   649 DCITLEGDQVSHRGALTGGYYDTRKSRLELQKDVRKAEEELGELEAKLNE  698

SMC3_silkworm/1-1203   699 TLRQELNKTEASINSIVSEMQRTETKQGKAKDIFDKVKADIRLMKEELAS  748
   SMC3_mouse/1-1217   699 NLRRNIERINNEIDQLMNQMQQIETQQRKFKASRDSILSEMKMLKEKRQQ  748

SMC3_silkworm/1-1203   749 IERFRGPKERSLAQCRSSLEAMQATKEGLESELHQELMEQLSTADQGKVD  798
   SMC3_mouse/1-1217   749 SEKTFMPKQRSLQSLEASLHAMESTRESLKAELGTDLLSQLSLEDQKRVD  798

SMC3_silkworm/1-1203   799 ELNDAIRRLTLENKEAFSARMNLEATKNKLENLLTNNLIRRKDELVQALQ  848
   SMC3_mouse/1-1217   799 ALNDEIRQLQQENRQLLNERIKLEGIITRVETYLNENLRKRLDQVEQELN  848

SMC3_silkworm/1-1203   849 EISVEDRKRRLANSKTDLSATEKRIKQINKELEEVERKVQAAVKNEKALK  898
   SMC3_mouse/1-1217   849 ELRETEGGTVLTATTSELEAINKRVKDTMARSEDLDNSIDKTEAGIKELQ  898

SMC3_silkworm/1-1203   899 LELDKWRNKEKEAQDKMEEDAKGLEKMASKEVLLQDKIQESLDKIAALGT  948
   SMC3_mouse/1-1217   899 KSMERWKNMEKEHMDAINHDTKELEKMTNRQGMLLKKKEECMKKIRELGS  948

SMC3_silkworm/1-1203   949 LPNAPELHAKYQKMSLKQLFKELEKANQHLKKYNHVNKKALDQFISFSEQ  998
   SMC3_mouse/1-1217   949 LPQ--EAFEKYQTLSLKQLFRKLEQCNTELKKYSHVNKKALDQFVNFSEQ  996

SMC3_silkworm/1-1203   999 KEKLYKRKEELDVGGEKIRELIETLEHRKLEAIQFTFKQVTKNFSEVFKK 1048
   SMC3_mouse/1-1217   997 KEKLIKRQEELDRGYKSIMELMNVLELRKYEAIQLTFKQVSKNFSEVFQK 1046

SMC3_silkworm/1-1203  1049 LVPQGRGSLIMRVATDEVQDVNP----------------ERANADQFAGV 1082
   SMC3_mouse/1-1217  1047 LVPGGKATLVMKKGDVEGSQSQDEGEGSGESERGSGSQSSVPSVDQFTGV 1096

SMC3_silkworm/1-1203  1083 GIKVSFTGGEGDMREMNQLSGGQKSLVALALIFAIQKCDPAPFYLFDEID 1132
   SMC3_mouse/1-1217  1097 GIRVSFTGKQGEMREMQQLSGGQKSLVALALIFAIQKCDPAPFYLFDEID 1146

SMC3_silkworm/1-1203  1133 QALDAQHRKAIANMIHELSSSAQFITTTFRPELLEHAHKFYGVKFRNKVS 1182
   SMC3_mouse/1-1217  1147 QALDAQHRKAVSDMIMELAVHAQFITTTFRPELLESADKFYGVKFRNKVS 1196

SMC3_silkworm/1-1203  1183 HVECVTQEEARDFVEDSATHA                              1203
   SMC3_mouse/1-1217  1197 HIDVITAEMAKDFVEDDTTHG                              1217
```
