# Supplementary material for: A molecular cell biology toolkit for the study of meiosis in the silkworm Bombyx mori
Source: G3 (Bethesda). 2023 Mar 13;13(5):jkad058. doi: 10.1093/g3journal/jkad058 (PMC10151401; doi:10.1093/g3journal/jkad058)
Supplement: jkad058_Supplementary_Data [file jkad058_supplementary_data.zip › Figure_S3_G3-2023-404089.docx]

TAAAAAAATACATTTCCCGCCATTTCAGCAATTGAAATTTAAATTAAAATCAATACATACATACAAATTTACATATTTCCTAAACGAAACGCACCACAAGCATACAAACAAACGGAAAAAATCTTTAAATCGTATACTAAAAAAAAACAAATTGAAAAACCTAAAG**ATG**GATATCTCTGATATTACGAAAGTAGCAAGCAGAAAACGAATCAGTGAAGATGCTGAAAAACTACGAGAAAAAGTTTCTCGTGAAACGGCGAAAATAATTAAATTAAAGGTGGCCCAGGACACGGCCTACTGGGACTTAAAAGAGAAACTACAACAAGTGGAAGCGAATCACGAACGCTTGCAACAGAACATGGTAGAAGTCCAGATGCAGCACGAGGCTACCTCCGGACAATACCAGGACGAACTGCGGCTCCGACCGGAGACTCTCAACAAAGTGCAGGGCGCGCGCCGCGTGTGCTCGGTGCTGGAGGAGGCGGCGGCGCGGCCGGGCCGCGCTCACCGCCCGCCGTGTGCAGTGGCGGCGAGCGAGGCGCGCGCGCGGCGGGCGCTGCAGGCGGCGCAGGAGGAGCGCGGGCGGGCGGAGCGGCGCGCAGAGGAGGCGCGGGCCCGGGAGGACGCCGAGAGGACGCTTCTACGGTGCAAACTACAGCAGAAAGAAGACTTCGTTGAAAATCTTCAAAAGGAAATCGAACGGTTGCAACAGGAGCTTCGAGATCGAATTGTTGAAGCCGACAATACTATTGAGAATTTGAAGCTAGGGTTGCAGCGGAGCGACGACGCGCGGGCGGCGCTGCAGCGGAGCCTGGCCCTCTCCGAGCAGCAGGGCGCCGAGCTGCGGGACCAGCACCAGCGTCTCCTGCTGCAGCTGCAGGACGCGCATGCTTCCCTCGAGGCATCATCGCGTGCTGTCGAAGAGCTCAAGGAACAAAACGACTCTCGGCAGCGAACCCTCGAGCGGCAGAACGAAGCCCTGGGCGCCGCCAGCGCGCAGGTGGAGCAGCTGACGGCGCGCGCCGCCGCGCTGGAGGACGAGAAGGCGGACGCGGTGTGCAGCCTGCGCGCCGAGCTCAGTGCGCTGCAGGCCCGCCACGACGACGTGCAGGGCCGGTTCCAGTCGTTCAGGGCGGAGAGCGAGGCGCGCGAGGCGGCGGCGGGCGCCAGCGTGGCGGCGCTCATGCGGGACGTGCAGGCCGAGGGCGAGCGCCGACACAAGCTGGAGGCCGCGCTGGGCGCCGCGCGAGACCTGGCCGCCCAGCGGGAGCGTCTGCTTGCGGACAAAGAAGACGAACTCAACAAACAGATGTCTATTATTCTGGATATGAGGAACGAAAAGGAGCGGCTCCAGGAGAAAATCCAGAGCATGCAGAACACCATCGACAACGTGCAGAAGGAGCTGTCGGGCCGCGGGGCGGGGCGGGGCGGCGCGCAGGGGGAGGCGCCGCAGCAGGACGCGCCCCCCCGCCCCCTCGACCCCGTGCTCTACAACTTCCTGAGCGACACCAGCCTGGACGCCGACGCGCTGGACAGCGACACCAGCCTGGACGCCGACGCGCTGGACGTATGCGTCACTGCTGCGTGTGAGTGTGTGCGCGTGGCGCGGGTGCTGACGAGCGAGGTGCGCCGCCGGTTCGAGGCGCTGTCCGGCGGTCGGCGAGCCGGCGCCGCCCAGCGTGCGCCCCGCCCCGCCCCGCAACATGCGTACCCTCAGTCGCCCGTGTCCTTGTCGCAAGTGAAGCTCGACTTGAAAACGAAACATAAATCATTCTTCAAAAACAAGAGAGTCGATTTGAAGACGAAGAAACTCAAG**TGA**CGGACTCGCTCTTATTCATGATTTATTCAAATTAAATATCACATTAATCCATCAAGAGATATTTTATTTCAAAATCTACTAAAATAAGGTATTATATAAATGTAAAAAATAAAAATAAAATCATAATGGAA
