# Supplementary material for: A molecular cell biology toolkit for the study of meiosis in the silkworm Bombyx mori
Source: G3 (Bethesda). 2023 Mar 13;13(5):jkad058. doi: 10.1093/g3journal/jkad058 (PMC10151401; doi:10.1093/g3journal/jkad058)
Supplement: jkad058_Supplementary_Data [file jkad058_supplementary_data.zip › Figure_S5_G3-2023-404089.docx]

**MDTNKLIKNVNNLRDYISVTENANSLKNSKIQFNMSTILALKKIRSFEIEYFNKHDENGFAIVLFQMMIN**

**LQPSKTWGVLGKELVNLLQYWLDAVRKHLISHNSHWWTFLKVLLKLLKDIRVKDPSLPNCLVENTAECLL**

**DLSTNSRPDAFQKLEILHCLNIICAESSREIRFALRNKFGNYFIKLASY**MSSCGYLPCQFVTLETLLRWL

VPRNQPELRATSAAYWFPEGMYSRQAVDIFLERPWMNFFQDARDFLNAHNSTNDLIVSVICQRFCIGEIM

LISGTEKQQSWLDINSTGRCLSVLLDPQLLGRFGPIDHKSCETVLITNDAAQTVVLKRDSSKPTISIQML

SPPQVHPSMISVGTSAPTHVELAVSARTDTRKLDHALRSVLADKYQLLLDLEHDPHPSPQSSVQNSKKMG

SSSEGDTRFSHPAEVRRKKRSGYVVRSNKPHSWKSPSTASTSSLAQLHDKLAALPLYKYDRDPVSVCAHP

ELSIVTEVSETDDRQSLNTASTLKFKPYGVCTRDNNNFDGIRKSQSDGELKTRSKDRRLSPAIHEGHSTS

CLLVATVGSADDSVINDTIERLSKSKDFNADKIVDLLVKEALHSNEIDSVNDSGINTDDKRNADANQESS

DAIDNTPFADAVREYQKINVNKKKGRVVISKSSTTDESNTDAISETNFVNSRRRTTKPTEEHSRVDEVKT

FDVEEVEEFFNQHFAQNAAGDVMISPTLAKKINESSSDSNDSFENYIVMNEINDNDNLNINDNDVIECLN

SMVDKVCSDFDKCTEYLTQSQMMLRFSDKGNDSQQHKDKDGKDKTSKKASKKGIKLKYKINTKTEKKVRQ

KNASKKTTSTIEDAVEDIDELKDKSNLLNNEKSKDFDRFPKIISRADFDLNDAQKDNLSNDNHISPIPNE

EAKRNSTPLQRRKRKLYSPKDETVERVRTPAILSDTEEDVTLSELKNTKKRPPKYTATSYKDIEKERKKQ

IRKPRNRRSKCTVETPSPRTLKLSRMFDKLKETVESNERIQLADRTSKRIEVYNFTSDSDDDFVMNKRTI

SKRNSATTVGSVESATTVRGKRPKKRINYNENKIGTGKREPKPKNVKPNKQSNKPTRMPLDRDLIDERMR

EAAPEELNTSLVIEQPQKEYSVPELVLKEPPKIEAIDEGNAKNEISKKRSKKKDLSSKTKLATGKLNTLE

VLSVGNRTESPLPNLVVESVPLKDEGNDSVSAKMLLKFKRIHEGQESRANDTTTTLNMLSDLEKNNTRET

HENVIDFENNLAHINEYFSGETDSDRSDISRKHGHLDQEKLCTAVVEDDNDIGMDISIATDGITGHGDED

EAPPSDKTHLQTRCLEIEDLDKTLKEYFEQLDKKINDIDDNSDNGNVINKSEIKNPIVRMTRLSSEDISK

VSRKSIESTCLSSKEKSPVVSICRIPSLEINKWLPSQSSKSKQNYLNDFTKQKSFKETKLYTNDEQSKDL

CKSLYDAHKWFPSRRESSTENSEKTEPNQNKVFIRELRLRSREISVVSSTDERKRKKTNRATDKDLKEHT

NDARPDDVSEVTNARRSMISPIKLGDFKSDANQRDRSQVDDCVKRIKDIFSQGKIVKSKLRALNTVSKDD

LAPDESVSDGRALSDCRKRKYEENENEVKRLRLDTVYVESSPSRSSVNEWFRRNEISSREETVNLSVLDT

VQNVLEKLDTTLVDANNNTSRKLVNLFVEAQKELSKQKEERRKMYKETASDILNAVVRLVDDKFADLDRR

SQEMETNFMERLKKQASEVIVNDCKQKRAMVQLLKEDFSSVVDHLNRNKI

ARMADILLO REPEATS PLECKSTRIN HOMOLOGY DOMAIN CLOSURE MOTIF COILED COIL
