# Supplementary material for: A molecular cell biology toolkit for the study of meiosis in the silkworm Bombyx mori
Source: G3 (Bethesda). 2023 Mar 13;13(5):jkad058. doi: 10.1093/g3journal/jkad058 (PMC10151401; doi:10.1093/g3journal/jkad058)
Supplement: jkad058_Supplementary_Data [file jkad058_supplementary_data.zip › Supplemental_Figure_Legends_G3-2023-404089.docx]

Supplemental Figure Legends

Figure S1: Protein alignment of SMC1 from silkworm and mouse. Comparison of silkworm and mouse SMC1 proteins show 51% identity and 70% positive substitutions. Default of 100% was used for colored shading of the conservation for residues in JalView.

Figure S2: Protein alignment of SMC3 from silkworm and mouse. Comparison of silkworm and mouse SMC3 proteins show 54% identity and 72% positive substitutions. Default of 100% was used for colored shading of the conservation for residues in JalView.

Figure S3: Potential gene sequence for *B. mori* *sycp1* identified in cDNA from testes. Potential start and stop codons are shown in bold.

Figure S4: Protein alignment of SYCP1 from silkworm and mouse. Default of 30% was used for colored shading of the conservation for residues in JalView.

Figure S5: Protein sequence of potential *B. mori* SYCP2 showing the difference in the two isoforms X1 and X2 of 1800 and 1611 aa, respectively. Isoform X2 is missing the sequence in bold. Underlined sequence is the predicted SYCP2 domain that contains the Armadillo repeats (green) and Pleckstrin homology domain (turquoise). The closure domain is shown in magenta, and the coiled coil domain is shown in yellow.

Figure S6: Lower magnified view of antibody staining in spermatocyte spreads showing various stages of meiosis. (A) Immunostaining of spermatocyte nuclei stained with SMC1 (yellow), SYCP2 (magenta) and SYCP1 (blue) in the merge with individual panels shown in white. All nuclei can be seen in the DAPI panel. (^) marks two examples of spermatocyte nuclei that are stained with SMC1 and not SYCP2 or SYCP1 and are likely spermatogonia or very early primary spermatocytes. Scale bar, 10 μm. (B) Immunostaining of spermatocyte nuclei stained with SYCP1 (yellow), SYCP3 (magenta) and SMC3 (blue) in the merge with individual panels shown in white. All nuclei can be seen in the DAPI panel. (^) marks two examples of spermatocyte nuclei that are stained with SMC3 and not SYCP1 or SYCP3 and are likely spermatogonia or very early primary spermatocytes. Scale bar, 10 μm. (C) Immunostaining of spermatocyte nuclei stained with SYCP1 (yellow), SYCP3 (magenta) and HOP1 (blue) in the merge with individual panels shown in white. All nuclei can be seen in the DAPI panel. Field shows many nuclei that are not stained with SYCP1, SYCP3 or HOP1 and are likely spermatogonia or very early primary spermatocytes. Scale bar, 5 μm. All image panels are projections.
