# Supplementary material for: A molecular cell biology toolkit for the study of meiosis in the silkworm Bombyx mori
Source: G3 (Bethesda). 2023 Mar 13;13(5):jkad058. doi: 10.1093/g3journal/jkad058 (PMC10151401; doi:10.1093/g3journal/jkad058)
Supplement: jkad058_Supplementary_Data [file jkad058_supplementary_data.zip › Table_S1_G3-2023-404089.docx]

Supplemental Table 1: Identification of potential homologs in *Bombyx mori* with protein identifier.

| Protein name | Protein function | Reference name | Source of protein | Bombyx mori identifier | Found by |
| --- | --- | --- | --- | --- | --- |
| SMC2 | Condensin | CAD59182.1 | mouse | XP_004924104.1 | blastp |
| SMC4 | Condensin | AAH61481.1 | mouse | XP_004933572.1 | blastp |
| CAP-H2 | Condensin | [NP_689512.2](https://www.ncbi.nlm.nih.gov/protein/NP_689512.2?report=genbank&log$=prottop&blast_rank=1&RID=U0SXPZUA01N) | human | XP_012549833.2 | blastp |
| CAP-D2 | Condensin | [NP_055680.3](https://www.ncbi.nlm.nih.gov/protein/NP_055680.3?report=genbank&log$=prottop&blast_rank=1&RID=U0T0MX2601N) | human | XP_012550994.3 | blastp |
| CAP-D3 | Condensin | [NP_056076.1](https://www.ncbi.nlm.nih.gov/protein/NP_056076.1?report=genbank&log$=prottop&blast_rank=1&RID=U0T5XS3601N) | human | XP_004925526.2 | blastp |
| CAP-G | Condensin | XP_037872351.1 | human | XP_037872351.1 | blastp |
| CAP-H | Condensin | [NP_056156.2](https://www.ncbi.nlm.nih.gov/protein/NP_056156.2?report=genbank&log$=prottop&blast_rank=1&RID=U0SK6G8C013) | human | XP_004931599.1 | blastp |
| SMC1* | Cohesin | YFL008W | yeast | XP_004923679 | Shinyapp |
| SMC3* | Cohesin | ENSG00000108055 | human | XP_004921724 | Shinyapp |
| RAD21 | Cohesin | NP_033035.3 | mouse | XP_037867555.1 | blastp |
| SA2 | Cohesin | NP_001345154.1 | mouse | XP_004927094.2 | blastp |
| PDS5 | Cohesin | YMR076C | yeast | XP_004922579  XP_037873907  XP_012550807 | Shinyapp |
| HOP1* | AE/LE | NP_001276461.1 | mouse | XP_021204681 | blastp |
| SYCP2* | AE/LE | See results |  | XP_037874109.1 | Multiple platforms |
| SYCP3* | AE/LE | NP_035647 | mouse | XP_004932505 | blastp |
| SYCP1* | Central Region SC | See results |  | See results | Multiple platforms |
| SINA | SC Regulator | FBgn0003410 | fly | XP_012552939 | Shinyapp |
| PCH2* | Meiotic Progression/Cell Cycle | NP_081458.1 | mouse | NP_001040375.1 | blastp |
| CDC7 | Meiotic Progression/Cell Cycle | ENSG00000097046 | human | XP_037871823 | Shinyapp |
| POLO | Meiotic Progression/Cell Cycle | FBgn0003124 | fly | XP_037871506  XP_037871507  NP_001296511 | Shinyapp |
| AURORA A | Meiotic Progression/Cell Cycle | ENSG00000087586 | human | XP_004930344 | Shinyapp |
| AURORA B | Meiotic Progression/Cell Cycle | ENSG00000178999 | human | NP_001274773.1 | blastp |
| MSP1 | Meiotic Progression/Cell Cycle | YGR028W | yeast | XP_004924407  XP_004924406 | Shinyapp |
| HUS1 | Meiotic Progression/Cell Cycle | ENSG00000136273 | human | XP_004924345  XP_021202432  XP_021202433  XP_021202434 | Shinyapp |
| BUBR1 | Meiotic Progression/Cell Cycle | OAP08383.1 | plants | XP_037868777.1 | blastp |
| CDK2 | Meiotic Progression/Cell Cycle | ENSMUSG00000025358 | mouse | NP_001266420 | Shinyapp |
| CDK4 | Meiotic Progression/Cell Cycle | ENSMUSG00000006728 | mouse | NP_001162052 | Shinyapp |
| SPO11 | DSB Formation | ENSG00000054796 | human | XP_012548355 | Shinyapp |
| RAD51 | DSB Repair | ENSG00000051180 | human | NP_001037484 | Shinyapp |
| DMC1 | DSB Repair | ENSG00000100206 | human | NP_001037552  XP_021207137 | Shinyapp |
| RAD54 | DSB Repair | ENSG00000085999 | human | XP_004928986 | Shinyapp |
| RAD50 | DSB Repair | ENSG00000113522 | human | XP_037868442 | Shinyapp |
| MRE11 | DSB Repair | ENSG00000020922 | human | NP_001036845 | Shinyapp |
| RPA1 | DSB Repair | YAR007C | yeast | NP_001036938 | Shinyapp |
| MCM8 | DSB Repair | ENSG00000125885 | human | XP_004930145  XP_037867101  XP_004930146 | Shinyapp |
| BLM1 | DSB Repair | NP_031576.4 | mouse | XP_037874493.1 | blastp |
| NABP2 | DSB Repair | XP_036011881.1 | mouse | XP_004927331.1 | blastp |
| MUS81 | DSB Repair | NP_082153.3 | mouse | XP_004921717.1 | blastp |
| RECQ5 | DSB Repair | FBgn0027375 | fly | XP_004925527.1 | blastp |
| BRAC2 | DSB Repair | P97929 | mouse | XP_037875235.1 | blastp |
| NBS1 | DSB Repair | NP_038780.3 | mouse | XP_004925881.2 | blastp |
| MSH4 | CO Promoting | ENSG00000057468 | human | XP_037868515 | Shinyapp |
| MSH5 | CO Promoting | ENSG00000204410 | human | XP_037866807 | Shinyapp |
| MLH1 | CO Promoting | ENSG00000076242 | human | XP_037876568  XP_037876569 | Shinyapp |
| MLH3 | CO Promoting | ENSG00000119684 | human | XP_037867421  XP_037867420 | Shinyapp |
| RNF212 | CO Promoting | NP_001349816.1 | mouse | XP_021207921.1 | blastp |
| HFM1/MER3 | CO Promoting | KAG2507485.1 | yeast | XP_037868302.1 | blastp |
| NARYA | CO Promoting | FBgn0031018 | fly | XP_037875478 | Shinyapp |

*Indicates antibody to *Bombyx mori* homolog has been made (this manuscript).
